# Compact representation of *k*-mer de Bruijn graphs for genome read assembly

**DOI:** 10.1186/1471-2105-14-313

**Published:** 2013-10-23

**Authors:** Einar Andreas Rødland

**Affiliations:** 1 Center for Cancer Biomedicine & Departement of Informatics, University of Oslo, 0316 Oslo, Norway; 2 Department of Tumor Biology, Institute for Cancer Research, The Norwegian Radium Hospital, Oslo University Hospital, 0424 Oslo, Norway

## Abstract

**Background:**

Processing of reads from high throughput sequencing is often done in terms of edges in the de Bruijn graph representing all *k*-mers from the reads. The memory requirements for storing all *k*-mers in a lookup table can be demanding, even after removal of read errors, but can be alleviated by using a memory efficient data structure.

**Results:**

The FM-index, which is based on the Burrows–Wheeler transform, provides an efficient data structure providing a searchable index of all substrings from a set of strings, and is used to compactly represent full genomes for use in mapping reads to a genome: the memory required to store this is in the same order of magnitude as the strings themselves. However, reads from high throughput sequences mostly have high coverage and so contain the same substrings multiple times from different reads. I here present a modification of the FM-index, which I call the kFM-index, for indexing the set of *k*-mers from the reads. For DNA sequences, this requires 5 bit of information for each vertex of the corresponding de Bruijn subgraph, i.e. for each different *k*−1-mer, plus some additional overhead, typically 0.5 to 1 bit per vertex, for storing the equivalent of the FM-index for walking the underlying de Bruijn graph and reproducing the actual *k*-mers efficiently.

**Conclusions:**

The kFM-index could replace more memory demanding data structures for storing the de Bruijn *k*-mer graph representation of sequence reads. A Java implementation with additional technical documentation is provided which demonstrates the applicability of the data structure (http://folk.uio.no/einarro/Projects/KFM-index/).

## Background

High throughput sequencing is generating huge amounts of sequence data even from single experiments. The raw sequence data will typically be too much to keep in the memory of most off-the-shelf computers, and with sequencing technologies progressing faster than the improvements in computer memory, the memory challenge is likely to increase in the future.

One key property of the raw sequencing data is that it is highly redundant. Genomes are usually sequenced at high coverage, which means there will frequently be at least 30–50 reads covering the same region of the genome, differing primarily by sequencing errors. Processing of sequencing reads for genome assembly usually involves two crucial steps: error correction to remove or correct sequencing errors, and assembly of overlapping reads to produce a smaller number of assembled sequences.

A common approach for simplifying the processing of the sequence data is to consider all the *k*-mers of the reads: i.e. all the *k*-substrings of the reads if we view them as strings. This set of *k*-strings is then thought of as a subgraph of the de Bruijn graph of order *k* − 1: i.e. one which has vertices corresponding to all *k* − 1-substrings and edges corresponding to the *k*-substrings. Even if sequenced at high coverage, each *k*-mer is thus represented only once, reducing the redundancy of the sequence data considerably. However, direct storage of all *k*-mers in a single list will require *k* letters per *k*-mer, i.e. 2*k* bit of information for DNA sequences, which can be quite memory consuming when *k* is large.

Naively, one might expect that this could be greately improved. From each vertex in the graph, there may be 4 possible out-going (or in-coming) edges if the graph represents DNA sequences: one for each of the nucleotides. Encoding which of these exist in the graph should require only 4 bit of information per vertex; if most vertices have only one out-edge, this might even be reduced towards 2 bit of information per vertex by only encoding which of the 4 possible edges is actually found. Of course, this approach requires that the vertices be known, but one might envision that the information about the vertices could be reconstructed when walking the graph: when walking *k*−1 steps, all *k*−1 letters of the resulting vertex will be known.

A traversable representation of the de Bruijn subgraph is equivalent to storing a searchable index of all the *k*-substrings. By traversable, I mean that it is possible to efficiently walk the graph starting at any vertex, to check if any *k*-mer or *k*−1-mer is present as an edge or vertex in the graph, and preferably also to be able to retrieve the *k*-mers and *k*−1-mers represented by the graph. Thus, it is not only important that the data structure be compact, but efficient algorithms for using it are just as important.

A number of data structures exist that provide more compact storage of the de Bruijn subgraph than naive *k*-mer lists or maps. Conway et al. [1] were able to represent a de Bruijn subgraph with 12 G edges in 40.8 GB, i.e. 28.5 bit per edge, by using a compressed array. Other approaches reduce memory by storing only a subset of the *k*-mers [2-4].

An entirely different approach uses a Bloom filter to store a hashed set of *k*-mers [5] using only 4 bit per *k*-mer. This is a probabilistic data structure with a known false positive rate, but where false positive edges can be identified by not being part of longer paths. However, while this data structure is effective for checking if a *k*-mer is contained in the graph, it does not easily allow listing of all vertices or edges. An enhancement of this method, Minia [6], avoids critical false positives and also allows retrieval of all vertices, but at the cost of higher memory consumption.

Another memory-efficient solution uses the FM-index [7], which is based on the Burrows–Wheeler transform [8] used to represent a suffix array [9], to store the collection of reads in a compressed form [10]. The Burrows–Wheeler transform was originally developed for text compression and has the property that recurrent substrings in the text before the transform result in single-letter repeats in the transformed string. The FM-index adds auxiliary information on top of the Burrows–Wheeler transformed sequence that effectively turns it into a compactly stored suffix array. When concatenating the reads, the coverage makes the Burrows–Wheeler transformed sequence dominated by single-letter repeats which are highly compressible [10]. Effectively, it requires 2 bit per edge to store the nucleotide, which corresponds to specifying the in-edge (or out-edge) of a vertex, and additional memory to store the run-length of the nucleotide, which corresponds to the *k*-mer count. At least up to 50 times coverage, this data structure should be able to store one edge per byte if used to represent the de Bruijn subgraph of *k*-mers.

It should be noted that the ability of different methods to handle read errors varies. Some of the cited methods are intended to perform error correction by filtering *k*-mers by their frequency, while other methods assume that read errors for the most part have been corrected or excluded in advance.

I here provide a data structure with strong similarities to the FM-index, but which stores the de Bruijn subgraph representing the *k*-mer substrings rather than entire sequences. It is based on the idea of storing for each vertex which of the possible in-coming edges are actually present. For each vertex it thus needs one bit of information per letter in the alphabet, i.e. 4 bit per vertex for DNA sequences, plus some additional data. The additional data consists of a grouping of vertices which requires one extra bit per vertex, plus the equivalent to the FM-index for mapping in-coming edges to their parent vertices. This version of the FM-index, which I call the *kFM-index* since it applies to an index of *k*-substrings, can be generated from the stored data, but for computational speed a subset of the index is kept in memory. All in all, a de Bruijn subgraph for DNA sequences, including the stored subset if the index, can be stored using 5–6 bits per vertex if memory consumption is critical. In the case where most vertices are of degree 1, i.e. have one in-edge and one out-edge, the stored data may be compressed down to approximately half the size.

Like the FM-index, the kFM-index stores only one strand of DNA sequences, and is suitable for walking the graph in one direction. For genome assembly, one does not know in advance which strand the read is on, and so normally are required to ensure that both the *k*-mers of the reads and their reverse complements are added to the graph. Some data structures, e.g. most hashing strategies, can combine *k*-mers and their reverse complements, and thus require roughly half the number of items. For the kFM-index, however, it is necessary to add both the reads and their reverse complements. In doing this, one may walk in the opposite direction by switching to the reverse complement, although there will be some computational overhead in doing so.

The basic operations available on the kFM-index are similar to those of the FM-index. Each vertex is identified by it’s index position, *i* = 0,1,…,*n*−1 where *n* is the number of vertices in the de Bruijn subgraph and the vertices are lexicographically ordered. For a given string, the vertices having that string as a prefix, identified by the interval of index positions, can be found efficiently: the computational time is proportional to the length of the string. Given a vertex, identified by it’s index position *i*, one can look up directly in the stored data which in-coming edges exist for that vertex. The index positions of the vertices from which the in-edges come can be computed efficiently. Thus, checking if a string exists as a path in the de Bruijn subgraph can be done. The reverse operation of identifying the string representation of a given vertex identified by index position *i* also exists, but is slower: time complexity is *O*(*k* lg*n*).

The kFM-index can be generated directly from a sorted list of in-edges, which is appropriate for amounts of sequence data that fit into the computer memory, although it should also be feasible to extend this by sorting the in-edges on disk: the time complexity is *O*(*Nk* lg*σ* lg*N*) where *N* is the total length of the sequence data, and thus the number of items to be sorted, and *Nk* lg*σ* is the amount of data being sorted. Generation of kFM-indexes in memory from sequentially read sequence data can be done by splitting the raw sequence data into parts, generate kFM-indexes for each part, and then perform pairwise merges of these kFM-indexes. The time complexity of generating the kFM-index in this manner is essentially *O*(*Nkσ* lg(*nm*)), where *n* is the number of vertices in the final de Bruijn graph (i.e. not counting identical *k*-mers), *σ* is the alphabet size, and *m* is the number of parts the initial sequence data is partitioned into. This has proven to be quite time consuming: in part because of the time complexity of the provided merge algorithm, but probably also in part due to an inefficient implementation. I expect that there is room for major improvements. In addition, these operations are all open to parallelisation.

Readers familiar with the FM-index will see the similarities to it, despite the fact that the FM-index represents all suffixes while this new data structure only stores information about *k*-substrings. Not only is the data structure very similar, but the functions and algorithms are also similar, or at least analogous, to those used with the FM-index. I therefore refer to this data structure as the kFM-index: an FM-index for *k*-substrings. And instead of pointing out the similarities throughout the article, I will point out differences where these are noteworthy.

A Java implementation of the data structure is provided as a demonstration.

## Methods

### Notation

Let Σ denote an *alphabet* of size *σ* = |Σ|, i.e. an arbitrary set whose elements we refer to as *letters*: for DNA sequences, Σ = {*A*,*C*,*G*,*T*} and *σ* = 4. A string of length *l*, or an *l*-string, is an element of *x* ∈ Σ^*l*^. Let $\Sigma^{*}=\cup_{l=0}^{\infty }\Sigma^{l}$ denote the set of all strings, including the empty string denoted *ε*. We denote the length of the string by |*x*|. If *x* and *y* are strings, *xy* denotes the concatenated string of length |*x*|+|*y*|; for sets *U* and *V* of strings, the set of concatenated strings is denoted *U*∘*V* = {*uv*|*u* ∈ *U*,*v* ∈ *V*}.

We write *x*<*y* to indicate that string *x* sorts lexicographically before *y* based on an ordering of the letters in Σ. In addition to the letters in Σ, we have two special characters *$* and *∞* with the properties that *$* <*a*<*∞* for all *a* ∈ Σ.

If *x* is an *l*-string, we write *x* = *x*_1_…*x*_*l*_ where *x*_*i*_ ∈ Σ are the letters. For *p*≤*q*, the [*p*,*q*] substring *x*_[*p*,*q*]_=*x*_*p*_…*x*_*q*_ is a string of length *q*−*p*+1: *x*_[*p*,*p*−1]_ is the empty string. A substring *x*_[1,*p*]_ at the start is referred to as a *prefix*, while a substring *x*_[*p*,*l*]_ at the end is referred to as a *suffix*. The operation of trimming away the last letter is denoted *x*^−^=*x*_[1,*l*−1]_=*x*_1_…*x*_*l*−1_.

For $S=(s_{1},\ldots ,s_{N})$ a list of strings, i.e. *s*_*i*_ ∈ Σ^∗^, let $S^{\left[k\right]}\subset \Sigma^{k}$ denote the set of length *k* substrings: i.e. *x* ∈ Σ^*k*^ is contained in $S^{\left[k\right]}$ if and only if there is a string s∈S with *x* = *s*_[*p*,*p*+*k*−1]_ for some position *p*.

We denote the base 2 logarithm by lg*x* = log2*x* which is convenient for quantifying information. Thus, the information required to specify one out of *n* options is lg *n* bit.

### Problem description

Given a set  of strings, e.g. a set of sequencing reads, we will construct a compact representation of $S^{\left[k\right]}$, i.e. the set of length *k* substrings, suitable for quickly checking if any particular *k*-string is present.

The data structure is best understood in terms of the *de Bruijn subgraph* representation of $S^{\left[k\right]}$. This has vertices $V=S^{\left[k-1\right]}$ and edges $E=S^{\left[k\right]}$ where *e* ∈ *E* is an edge from *e*_[1,*k*−1]_ to *e*_[2,*k*]_. It is a subgraph of the de Bruijn graph of order *k*−1, i.e. with vertices Σ^*k*−1^ and edges Σ^*k*^. Some authors may refer to this as a *word graph*, or even just a *de Bruijn graph*. Since the set of vertices can be deduced from the edges, storing $S^{\left[k\right]}$ is effectively the same as storing the information encoded in the de Bruijn subgraph. However, the graph structure highlights the overlap between edges meeting at vertices.

While some authors focus on *k* as the length of the strings represented by the edges, others focus on the order of the graph which is the *k*−1 length of the strings represented by the vertices. Since our purpose is to represent the *k*-mer composition of the sequences, it is natural to focus on *k* as the *k*-mer length. However, the implementation of the algorithms is more naturally centered around the vertices, and so the Java implementation focuses on the order of the de Bruijn subgraph which is *k*−1.

### The kFM-index data structure

The data structure for storing the *k*-substrings $S^{\left[k\right]}$ from a set of strings  has similarities to the FM-index and the Burrows–Wheeler transformation. One similarity is that the data structure stores the prefixing letters, which represent the in-edges to vertices, and backtracks the de Bruijn subgraph through these in-coming edges rather than walking paths from beginning to end; the sequences, including the strings the vertices and edges represent, are thus reconstructed from the in-edge data when backtracking through the graph.

**Figure 1:**
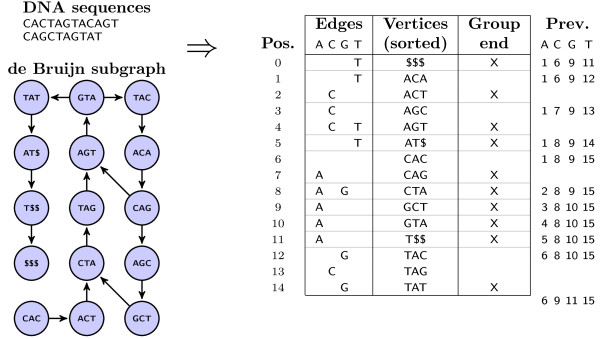
**The kFM-index data and corresponding de Bruijn subgraph.** Representation of the data structure for DNA 4-mers. The *vertex strings*, lexigographically sorted, are not stored, but reconstructed from the edge and group end data. The *edges* columns indicate in-coming edges to each vertex, i.e. letters that may prefix the vertex strings. The *group end flag* inidicates groups of vertices with the same *k*−2-prefix. The *previous position* data can be generated from the edge set data and group end data and is constant within each vertex group; a subset is stored for computational speed.

The initial de Bruijn subgraph representing the *k*-string composition $S^{\left[k\right]}$ may contain any number of *finalvertices*: i.e. vertices for which there are no out-going edges. These final vertices correspond to *k*−1-strings found only as suffixes of the strings in , and represent a problem as they cannot be reached by backtracking the de Bruijn subgraph. As the data structure does not store the *k*−1-strings for each vertex, but instead reconstructs these strings when walking the graph, these final vertices cannot be thus reconstructed. The solution is to add extra vertices and edges leading from these final vertices to a special final vertex from which we may start the reconstruction. See Figure 1 for an example.

Let $V_{\text{final}}\subset S^{\left[k-1\right]}$ be a set that includes all *k*−1-strings which are final vertices in the graph with edge set $S^{\left[k\right]}$: i.e. if $v\in S^{\left[k-1\right]}$ and *v* is not a prefix of any string in $S^{\left[k\right]}$, then *v* has to be in *V*_final_. Ideally, in order to get the most compact representation of $S^{\left[k\right]}$, we want *V*_final_ to contain only these strings. However, we might start off by letting *V*_final_ contain all *k*−1-suffixes of the strings in , knowing that the vertices required to be in *V*_final_ have to be a subset of these, and then later prune away superfluous edges and vertices. Hence, we permit *V*_final_ to be bigger than strictly required.

We now define the *final-completed* de Bruijn subgraph with paths added from each *v* ∈ *V*_final_ to a special vertex, *$*^*k*−1^=*$*…*$* which we refer to as *the final vertex*, having vertices

(1) $$V=S^{\left[k-1\right]}\cup {\left(V_{\text{final}}\circ {\$}^{k-1}\right)}^{\left[k-1\right]}\cup \{{\$}^{k-1}\}$$

and edges

(2) $$E=S^{\left[k\right]}\cup {\left(V_{\text{final}}\circ {\$}^{k-1}\right)}^{\left[k\right]}$$

where *V*_final_∘*$*^*k*−1^ = {*v$*…*$* ∣ *v* ∈ *V*_final_} denotes the strings from which these additional paths are constructed. These are strings over an extended alphabet Σ∪{$}, where *$* is a special character that is sorted before any of the letters of Σ. The added vertices, i.e. those containing one or more *$* at the end, are referred to as *final-completing vertices* and are parts of paths leading to the final vertex. In fact, the final-completing vertices form a tree with the final vertex, *$*^*k*−1^, as the root. Note that, for the case where *V*_final_ is empty, we explicitly add the special vertex *$*^*k*−1^: this is purely a matter of convenience.

By this extension of the de Bruijn subgraph, we have ensured that there is exactly one final vertex that cannot be reached by backtracking the graph, namely the final vertex *$*^*k*−1^. When sorting the vertices lexicographically, this will always come first. Note that we do not require that the final vertex be reachable from the rest of the graph. If the original graph had *V*_final_ empty, this would be the case.

We may identify *E* with a subset of Σ×*V* describing the set of in-coming edges to each vertex, and will by abuse of notation say that the pair (*a*,*v*) ∈ Σ×*V* is an edge if the concatenation *av* ∈ *E*. We denote the in-coming edges to *v* by *E*_*v*_⊂Σ: i.e. *E*_*v*_ = {*a* ∈ Σ ∣*av* ∈ *E*}.

Note that backtracking through this de Bruijn subgraph corresponds to reading the strings in the backwards direction, from the end of the string towards the beginning, just as with the FM-index. A variant of the data structure which naturally reads the strings in the forward direction can be obtained by performing the construction on the reversed strings, the only effect of which is on the sorting of strings in the index which would then be based on the reversed string.

#### Main data

Let *n* = |*V*| be the number of vertices of the final-completed de Bruijn subgraph, and let *v*_0_,…,*v*_*n*−1_ denote the vertices of *V* in lexicographic order; in particular, *v*_0_ = *$*^*k*−1^, which is the only final vertex of the final-completed de Bruijn subgraph. The basic information required to store the final-completed de Bruijn subgraph of $S^{\left[k\right]}$ is: 

Edges: The set $E_{v_{i}}\subset \Sigma$ of edges from each vertex *v*_*i*_ is stored; i.e. the edge set *E* identified as a subset of Σ×*V*. This may be encoded as a *σ*×*n* array, *η*(*a*,*i*), with binary values: i.e. *η*(*a*,*i*)∈{*false*,*true*} indicates if *av*_*i*_∈*E*. The in-edges *E*_*i*_={*a*∣*av*_*i*_∈*E*} to vertex *v*_*i*_ may be represented as a bit-mapped number on which set operations correspond to binary operations.

Group end flags: We group vertices *v* ∈ *V* with the same *k*−2-prefix together: i.e. *u* and *v* are grouped together if *u*^−^ = *u*_[1,*k*−2]_ and *v*^−^ = *v*_[1,*k*−2]_ are identical. We indicate the group end by a flag *f*_*i*_ which is *true* if *v*_*i*_ is the last vertex in its group, *false* otherwise. This requires one bit of information per vertex.

More formally, these binary arrays take logical values, *true* or *false*, as defined by

(3) $$\eta (a,i)\overset{\text{def}}{\Leftrightarrow }a\in E_{v_{i}}$$

and

(4) $$f_{i}\overset{\text{def}}{\Leftrightarrow }v_{i}^{-}\neq v_{i+1}^{-}\text{or}i=n-1$$

where *v*_*i*_<*v*_*i*+1_ follows from the lexicographic sorting of the vertices. We could have added as a convention that *v*_*n*_=*∞*^*k*−1^, in which case special handling of the final position would not have been required.

The grouping of vertices with the same *k*−2-prefix allows us to check which in-edges originate from the same vertices: for *a*,*b* ∈ Σ, *u*,*v* ∈ *V*, edges *au* and *bv* originate from vertices *au*^−^ and *bv*^−^ respectively, which is the same vertex if *a* = *b* and *u*^−^=*v*^−^, which corresponds to checking if *u* and *v* are in the same vertex group.

#### Index to previous vertex position

In addition to the main data, there is an indexing function which may be generated from the main data. These are values per vertex group, i.e. constant within the groups of vertices with the same *k*−2-prefix. Storing the entire indexing function as auxiliary data would take a lot of memory, while computing everything from scratch would take a lot of time. Instead, a balance between memory and speed is obtained by storing a sparse subset, e.g. at regular intervals, and recompute the values in-between on demand. This index corresponds to the FM-index and provides a map from a vertex to the origin vertex of its in-edges.

For each letter *a* ∈ Σ, let *τ*(*a*) denote the number of vertex groups that contain an *a* in-edge. For *i* = 0,…,*n*, let *c*(*a*,*i*) denote the number of vertex groups prior to position *i* that contain an *a* in-edge: the vertex group containing *v*_*i*_ is not included in this sum. This makes *τ*(*a*) = *c*(*a*,*n*), since the vertices all have positions *i*<*n*.

For *a* ∈ Σ, *i* = 0,…,*n*, let

(5) $$\rho (a,i)=1+\underset{b<a}{\sum }\tau (b)+c(a,i)$$

where the summation of *b* <*a* is for all *b* ∈ Σ lexicographically prior to *a*, and the 1 corresponds to skipping the vertex *v*_0_ = *$*^*k*−1^. Note that if *a*^′^ is the letter following *a* in the alphabet Σ, i.e. *a*^′^ = *a*+1 if we consider the letters to be enumerated 0,…,*σ*−1, then *ρ*(*a*^′^,0) = *ρ*(*a*,*n*); and for *a* the last letter of Σ, i.e. *a* = *σ* − 1, we have *ρ*(*a*,*n*) = *n*.

The position array, *ρ*(*a*,*i*), has the property that if vertex *v*_*i*_ has an in-coming edge *av*_*i*_∈*E*, i.e. $a\in E_{v_{i}}$, this edge comes from vertex *v*_*ρ*(*a*,*i*)_. A more general definition is that

(6) $$\rho (a,i)=\min\{j|v_{j}\geq {\text{av}}_{i}^{-}\text{or}j=n\}$$

where, as before, *v*^−^ = *v*_[1,*k*−2]_ for *k*−1-strings *v* ∈ *V* and *u*≥*v* refers to the lexicographic ordering of strings.

We may also note that, if we represent letters *a* by integers 0,…,*σ*−1, we have *ρ*(*a*,*n*) = *ρ*(*a*+1,0), and may define *ρ*(*an*+*i*) = *ρ*(*a*,*i*), where *ρ*(*j*) is again a non-decreasing function for j=0,…,n×σ with *ρ*(0)=1 and *ρ*(*n*·*σ*)=*n*. Representing *ρ*(*a*,*i*) in terms of *ρ*(*an*+*i*) is sometimes convenient, e.g. when we want to compute the inverse, and similarly we may use *an*+*i* to represent an in-edge, or potential in-edge, (*a*,*v*_*i*_).

As may be noted, the role of *ρ*(*a*,*i*) in mapping from one vertex to another is essentially the same as the FM-index for mapping from one suffix to another. The main difference is in the grouping of vertices into groups, where counts are over vertex groups rather than individual vertices. The reason this vertex grouping is required is that the de Bruijn subgraph allows branching, i.e. for vertices to have more than one out-edge; the vertices in a vertex group share the same set of in-edges. The traditional FM-index could be envisioned as a de Bruijn subgraph with no branching, where each vertex has exactly one in-edge and one out-edge, and so the vertex groups would all consist of just one vertex.

#### Auxiliary data stored for computation speed

Storing *ρ*(*a*,*i*) (or equivalently *c*(*a*,*i*)) as an array of integers requires *σ*×lg *n* bit for each vertex. However, if *ρ*(*a*,*j*) are known for some nearby *j*, the number of computational steps to compute *ρ*(*a*,*i*) from *ρ*(*a*,*j*) is proportional to |*i*−*j*|. So by storing only a subset of the *ρ*(*a*,*i*), e.g. every *q*th position, the memory required for storing the auxiliary data is greatly reduced, but at the cost of computational time for determining *ρ*(*a*,*i*). The partial storing of *ρ*(*a*,*i*) is essentially the same as for the FM-index, and can be done in a number of different ways.

Let 0=*i*_0_<⋯<*i*_*ζ*_=*n* be the position for which *ρ*(*a*,*i*) is to be stored, with *i*_*r*_−*i*_*r*−1_≤*q* for some chosen *q*. The stored values then consist of an array *κ*(*aζ*+*r*)=*ρ*(*a*,*i*_*r*_) where *a* = 0,…,*σ*−1 represent the letters. Thus, we have *κ*(*j*) for 0≤*j*≤*σζ* with increments 0≤*κ*(*j*)−*κ*(*j*−1)≤*q*. Storing the entire *κ* array is, however, still space consuming unless *q* is allowed to be big, in which case computing *ρ*(*a*,*i*) will take time.

Knowing that *κ*(*j*) increases by values between 0 and *q*, we can write *κ*(*j*)=*u*_*j*_+*qU*_*j*_ where *U*_*j*_=⌊*κ*(*j*)/*q*⌋ and 0≤*u*_*j*_<*q* and store the *u*_*j*_ in a bit-packed array. The values *U*_*j*_ now have increments *ΔU*_*j*_=*U*_*j*_−*U*_*j*−1_∈{0,1}. We store the increments *ΔU*_*j*_ as an array of bits, and a subset of the *U*_*j*_ from which the remaining *U*_*j*_ can then be computed efficiently. This allows us to select a much smaller value for *q* than would otherwise be feasible.

#### Java implementation

The Java implementation stores *η*(*a*,*i*) and *f*_*i*_ as a *σ*+1 bit block for each vertex *i*. These blocks are then packed into 64 bit words (long integers). For DNA sequences, each 64 bit word thus stores 12 vertices, each using 5 bit.

The stored values of *ρ*(*a*,*i*) are expressed in terms of *κ*(*j*)=*u*_*j*_+*qU*_*j*_. The *u*_*j*_ are bit-packed into an array to preserve memory. For reconstruction of the *U*_*j*_, every 64th *U*_*j*_ is stored, i.e. *U*_64*r*_, and the increments *ΔU*_*j*_ are stored in blocks of 64 bits. Any *U*_*j*_ can then be computed efficiently from *U*_64*r*_, *r* = ⌊ *j*/64⌋, and the increments *ΔU*_64*r*+1_,…,*ΔU*_*j*_ using operations on 64 bit words.

### Fundamental functions for utilising the data structure

For a string *x* and *a* ∈ Σ, we define the function *γ*(*x*) recursively by

(7) $$\begin{array}{l} \gamma (\varepsilon )=\gamma (\$)=0,\;\gamma (\infty )=n,\\ \gamma (a)=\rho (a,0),\;\gamma (\text{ax})=\rho (a,\gamma (x)). \end{array}$$

This function has a natural interpretation. If |*x*|<*k*,*γ*(*x*) is the smallest non-negative integer *i* for which *x*≤*v*_*i*_, or *i* = *n* if none such exists. If |*x*|≥*k*,*γ*(*x*) is the smallest integer *i* for which *x*≤*v*_*i*_*y* for some string *y* ∈ Σ^∗^ so that *v*_*i*_*y* can be realised as a path in the de Bruijn subgraph (*V*,*E*), i.e. all *k*-substrings of *v*_*i*_*y* are in *E*, or *i* = *n* if no such string exists. This property is formally proved in Lemma 2 in the Proofs of results appendix.

In order to utilise the data structure, for strings *x* with length |*x*|<*k*, we define two utility functions: the first vertex *v* ≥ *x* is

(8) $$\alpha (x)=\gamma (x)=\min\{i|v_{i}\geq x\text{or}i=n\}$$

while the first vertex with *v*>*x∞* is

(9) $$\beta (x)=\gamma (\mathrm{x\infty})=\min\{i|v_{i}>\mathrm{x\infty}\;\text{or}\;i=n\}$$

Recall that *∞* >*a* for all *a* ∈ Σ, so *β*(*x*) for *x* ∈ Σ^*l*^, *l*<*k*, finds the first *v* ∈ *V* for which *v*_[1,*l*]_>*x*, while *α*(*x*) finds the first *v* for which *v*_[1,*l*]_≥*x*. Thus, *α* and *β* are defined by the property

(10) $$\left\{v\in V|v_{\left[1,l\right]}=x\}=\{v_{i}|\alpha (x)\leq i<\beta (x)\right\}$$

for all *x* ∈ Σ^*l*^, *l*<*k*, making them exactly the functions we need to identify all vertices starting with a given prefix.

Note that if we add the vertex *v*_*n*_=*∞*^*k*−1^ to the list, we wouldn’t have to specify the *i* = *n* case in the above definitions. Again, this addition would purely be a matter of convenience and not have any practical impact.

### Algorithms for using the data structure

The above described data structure encodes a de Bruijn subgraph representation of the *k*-substring composition of the strings . However, to utilise this representation, we need efficient algorithms.

Throughout the algorithms, vertices of *V* will be identified by their position *i* ∈ {0,…,*n*−1} in the lexicographically sorted list *v*_0_,…,*v*_*n*−1_ where *n* = |*V*|. The string that each vertex represents will generally not be known.

The alphabet Σ is known from the start and the letters ordered. Computationally, it is natural to represent the letters by numbers 0,…,*σ*−1 (ignoring the letter *$*) since they are to be used as array indexes. However, for added readability, I will denote them as letters *a* ∈ Σ in the algorithms rather as numerical indexes.

#### Computing the previous position ***ρ(a,i)*** for arbitrary positions

A first step is to be able to compute *ρ*(*a*,*i*) for arbitrary positions based on the stored data.

Let 0=*i*_0_<…<*i*_*ζ*_ = *n* be the values for which *ρ*(*a*,*i*) is stored, i.e. *ρ*_store_(*a*,*i*_*r*_)=*ρ*(*a*,*i*_*r*_), and define functions

(11) $$\iota^{+}(i)=\min\{i_{r}|i_{r}\geq i\},\;\iota^{-}(i)=\max\{i_{r}|i_{r}\leq i\}$$

for pointing to the next or previous stored value. We may then compute *ρ*(*a*,*i*) by aggregating from the vertex group containing *ρ*(*a*,*ι*^−^(*i*)) as in Algorithm 2.

**Algorithm 1** Compute arbitrary *ρ*(*a*,*i*) from previous stored value

An alternative is to start at *ρ*(*a*,*ι*^+^(*i*)) and subtract contributions from vertex groups prior to this position, which can be done with a similar algorithm (see Additional file 1). To speed up the procedure, one may identify the nearest stored value, either previous or later, and use whichever of the two algorithms is appropriate. This will on average double the speed, and is done in the Java implementation.

#### Find all vertices with a particular prefix

The functions *α*(*x*) and *β*(*x*) for strings *x* with length |*x*|<*k* are both naturally expressed in terms of the the function *γ*. We note that *γ* gets called either as *γ*(*x*) = *γ*(*x$*) or as *γ*(*x∞*), and so we can express these as *γ*(*x*)=*γ*(*x$*) = *γ*(*x*,0) and *γ*(*x∞*) = *γ*(*x*,*n*) where *γ*(*ax*,*i*)=*ρ*(*a*,*γ*(*x*,*i*)) and for *x* = *ε* the empty string *γ*(*ε*,*i*)=*i*. Algorithm 2 details the computations.

**Algorithm 2** Algorithm for computing *γ*(*x*,*i*): then, *α*(*x*)=*γ*(*x*)=*γ*(*x*,0) and *β*(*x*)=*γ*(*x∞*)=*γ*(*x*,*n*)

By combining the call to *α*(*x*) and *β*(*x*) into one function (Algorithm 3), it is possible to exploit the fact that when the interval is empty the two computations become identical. The return value [ *α*(*x*),*β*(*x*)〉 represents the interval *α*(*x*),…,*β*(*x*)−1, and may be represented by a pair (*α*,*β*). If *α*(*x*)=*β*(*x*), the interval is empty: the function could be modified to abort once it is clear that the resulting interval will be empty, or at least reduce computations by half once it is clear that *α*(*x*)=*β*(*x*).

**Algorithm 3** Compute [*α*(*x*),*β*(*x*)〉

When merging two kFM-indexes, [ *α*(*x*),*β*(*x*)〉 is computed numerous times on one kFM-index with *x* being vertices (or vertex prefixes) from the other kFM-index. When the result maps a vertex in one to an vertex in the other kFM-index, the two are merged; when a vertex *v* is mapped to an empty interval in the other kFM-index, the position *α*(*v*)=*β*(*v*) tells the merge procedure into which position it should be merged.

#### Backtracking through the de Bruijn subgraph

For a string *x* of length |*x*|=*l*≥*k*, walking the de Bruijn subgraph path corresponding to *x* is most easily done by starting at the end of *x* and backtracking the graph towards the start of *x*. Algorithm 4 provides the algorithm for doing this: it will start at the *k*−1-suffix *x*_[*l*−*k*+2,*l*]_ and backtrack one step at a time, exiting if the string leaves the graph.

**Algorithm 4** Backtrack the de Bruijn subgraph for a string *x* ≥ *k*

#### Identifying the string value of a vertex

If we start with a vertex identified by its position *i*, we can determine the string that vertex represents. In order to do so, we need a function *ρ*^inv^:{0,…,*n*−1}→Σ×{0,…,*n*−1} which has the property that

(12) $$\rho^{\text{inv}}(i)=(a,j)\;\Leftrightarrow \;\rho (a,j)=i,\rho (a,j+1)=i+1$$

and where *ρ*^inv^(0)=(*$*,0). The pair (*a*,*j*) can be found through a binary search. However, since the computation of *ρ* is done in a stepwise manner starting at one of the stored values, a binary search should be performed on the stored values to identify the interval that contains the solution, and the stepwise procedure then followed until the solution is found.

The interpretation of *ρ*^inv^(*i*)=(*a*,*j*) is that *a* is the first letter of *v*_*i*_, while *j* is the last vertex in the vertex group with *k*−2-prefix equal to the *k*−2-suffix of *v*_*i*_: i.e. there is an edge from *v*_*i*_ to a vertex in the same vertex group as *v*_*j*_. However, since the vertices in the same vertex group only differ by the last letter, and we don’t have to determine this letter, we do not have to determine which vertex (or vertices) in this vertex group has an edge from *v*_*i*_. By iterating this procedure *k*−1 times as in Algorithm 5, we can find the string *v*_*i*_.

**Algorithm 5** Return string *v*_*i*_ for position *i*

### Generating the kFM-index from a set of strings

The simplest way to generate the main data, i.e. the in-edge list and vertex group end flags, from a set of strings, is to generate the set of all *k*-substrings, including the final-completing strings with one or more more *$* attached at the end, sort and group them by their *k*−1-suffix, and then generate the in-edge list and group end flags directly from this. Once the main data, i.e. the binary arrays *η* and *f*_*i*_ representing the edge set *E* and the group end flags, have been generated, the auxiliary data can be generated from these.

This brute force approach requires a fair amount of memory since all *k* letters need to be stored for all *k*-substrings. The list of *k*-substrings thus takes up *k* times as much memory than the original strings from which they are generated, and so is only feasible when the original string data is moderate in size.

If the string data is large, so that not all *k*-substrings of $S^{\left[k\right]}$ can be kept in memory, the job may be split up. The set  of strings may be split up into smaller subsets, the kFM-index generated for each subset, and pairwise merging of kFM-indexes may then be performed to combine the subset based indexes into a kFM-index for the whole set.

Note that this procedure will generate a full set of final-completing vertices, i.e. those containing *$* at the end, even when they are not required by the kFM-index. We may reduce the kFM-index by checking which final-completing vertices are actually required in order to be able to reach the entire graph by backtracking from the final vertex. However, even if we do this for the initially generated kFM-indexes so as to ensure these contain minimal sets of final-completing vertices, when we merge the kFM-indexes for the string subsets, superfluous final-completing vertices may again occur when final-completing vertices required in one kFM-index are rendered superfluous by the edges of the other kFM-index. Hence, we may wish to prune away these final-completing vertices at the end, or in some of the intermediary merges.

#### Merging two kFM-indexes

If we have two kFM-indexes denoted *A* and *B*, one with *n*_*A*_ elements and the other with *n*_*B*_ elements, these can be merged in two steps. First, we merge the two lists into a list of length *n*_*A*_+*n*_*B*_. In the process of merging the two lists, rows representing the same vertex are not combined, but instead we mark the occurences where one vertex merged list is identical to the next one so that these may later be combined. In addition, vertex groups found in both *A* and *B* must be merged into one vertex group, which involves removing the group end flag from any vertex not at the end of the merged vertex group. After that, we sequentially pass through the *n*_*A*_+*n*_*B*_-length merged list, combining identical vertices into one vertex.

Instead of generating the *n*_*A*_+*n*_*B*_-length list in full, which would require the same amount of memory as the two original kFM-indexes of length *n*_*A*_ and *n*_*B*_, we can represent the merge by a *n*_*A*_+*n*_*B*_ bit array indicating which of the two lists go into each position. Another bit array is used to mark identical vertices, which only needs *n*_*A*_ bit since we only need to store which vertices in *A* are also found in *B*. Finally, we use an *n*_*A*_+*n*_*B*_ bit array to mark vertices in the merged list that should not keep whatever group end flag it might have: this is required for vertex groups found in both *A* and *B* to ensure that only the final vertex in the group retains it group end flag.

**Algorithm 6** Merge two kFM-indices

It is sufficient to find the positions in the *n*_*A*_+*n*_*B*_-list of the *n*_*A*_ vertices from the kFM-index *A*. We may assume *n*_*A*_≤*n*_*B*_ since that will require only *n*_*A*_ lookups; the *n*_*B*_ vertices from the kFM-index *B* will then be in the remaining positions. To do this, for all items *i* = 0,…,*n*_*A*_−1 in the *A* list, compute the string $v_{i}^{A}$ of that vertex. We then look up the position of $v_{i}^{A}$ in the kFM-index *B* by computing $[\alpha_{B}(v_{i}^{A}),\beta_{B}(v_{i}^{A})\rangle$. If the interval is empty, i.e. $\alpha_{B}(v_{i}^{A})=\beta_{B}(v_{i}^{A})$, the vertex goes into position $i+\alpha_{B}(v_{i}^{A})$ and so we mark this position as containing a vertex from *A*. If the interval is not empty, the vertex $v_{i}^{A}$ is found in position $\alpha_{B}(v_{i}^{A})$ in the *B* list (and $\beta_{B}(v_{i}^{A})=\alpha_{B}(v_{i}^{A})+1$), and so again we mark position $i+\alpha_{B}(v_{i}^{A})$ as containing an *A* vertex, while the vertex from *B* takes position $i+\alpha_{B}(v_{i}^{A})+1$; in addition, we mark vertex *i* in the *A* list as having a duplicate in the *B* list.

We similarly need to iterate over all vertex groups in *A*, i.e. all non-empty [ *α*_*A*_(*u*),*β*_*A*_(*u*)〉 for *u* ∈ Σ^*k*−2^. If the vertex groups [ *α*_*A*_(*u*),*β*_*A*_(*u*)〉 and [ *α*_*B*_(*u*),*β*_*B*_(*u*)〉 are both non-empty, we know that the vertices in the interval [ *α*_*A*_(*u*)+*α*_*B*_(*u*),*β*_*A*_(*u*)+*β*_*B*_(*u*)〉 correspond to the *u* vertex group in the preliminary merged list. The last vertex, i.e. the one in position *β*_*A*_(*u*)+*β*_*B*_(*u*)−1, will have its group end flag set from either list *A* or *B*. However, there will be another vertex with its group end flag set from the other list which may be in any of the other positions in the interval, and to ensure that this is unflagged, we mark positions *α*_*A*_(*u*)+*α*_*B*_(*u*),…,*β*_*A*_(*u*)+*β*_*B*_(*u*)−2 for group end flag removal.

Rather than process the items *i* = 0,…,*n*_*A*_−1 sequentially, which requires computing VERTEX(*i*) for each and then looking these up in *B*, it is more efficient to recurse over all *p*-mers for *p* = 1,…,*k*−1, each recursion adding all possible one letter prefixes. Thus, *l* = 0 corresponds to vertices, *l*=1 to vertex groups, [ *α*,*β*〉 refers to index intervals corresponding to a given *p*-mer prefix in *A* or *B*. The function PREMERGE performs this recursion. It is called from MERGE where *l*=*k*−1−*p* is the number of trailing *$*.

**Algorithm 7** Prepare merge: recurse over *A* intervals

Once we have the *n*_*A*_+*n*_*B*_ bit array indicating which positions in the *n*_*A*_+*n*_*B*_ merge comes from *A* or *B*, another *n*_*A*_ bit array telling which vertices in *A* are also found in *B*, and a third *n*_*A*_+*n*_*B*_ bit array marking vertices for group end flag removal, we can merge the two lists sequentially using PERFORMMERGE.

**Algorithm 8** Merge subroutine: perform merge based on merge information

The three bit arrays used to facilitate the merge require 3*n*_*A*_+2*n*_*B*_ bit of extra data. The merge requires *n*_*A*_ lookups to find $v_{i}^{A}$ and then $[\;\alpha_{B}(v_{i}^{A}),\beta_{B}(v_{i}^{A})\rangle$ followed by the copying of all *n*_*A*_+*n*_*B*_ elements combining them into one when they represent the same vertex. In addition, the creation of the target list requires a temporary duplication of all the vertex and edge data, but this could be avoided by using a data structure in which the merged list is being created gradually as needed while memory used by *A* and *B* is gradually released as they are being merged. The Java implementation provided does this.

#### Pruning away superfluous final-completing vertices

The removal of superfluous final-completing vertices, i.e. vertices ending with one or more *$* characters that are not required in order to avoid final vertices that have no out-edge, can be done by a few simple rules. We can perform these checks by a recursive approach, exploiting that the final-completing vertices form a tree with the final vertex, *$*^*k*−1^, as the root. We start at the final vertex, which is in position 0 of the kFM-index, and recursively backtrack through all in-edges at most *k*−2 steps to reach all final-completing vertices in *V*, performing the tests depth-first. We then identify edges and vertices that can be removed. After all final-completing vertices have been processed in this manner, we condense the list by removing the superfluous edges and vertices from the list.

**Algorithm 9** Prune the index of unneeded final-completing vertices

If *v* ∈ *V* is a final-completing vertex, it is superfluous and can be removed if it has no in-edges. By having no in-edges, I include cases where the in-edges from vertices marked for exclusion have been removed: this is the reason why the tests must be done depth-first. Since *ρ* depends on which in-edges exist in each vertex group, superfluous in-edges can be removed immediately only for final-completing vertices where there is another vertex in the same vertex group with an in-edge from the same vertex (i.e. the same in-edge prefix). In general, edges and vertices must be marked for removal while the final-completing vertices are checked, and only removed after the checking is finished.

If *v* = *u$* ∈ *V* is a final-completing vertex ending with a single *$* and *e* = *av* is an in-edge to *v* for some *a* ∈ Σ, the edge *av* can be removed if there is an *a*-in-edge to another vertex in the same vertex group as *v*: i.e. if there is another vertex *ub* ∈ *V* which has an in-edge *aub* ∈ *E*, the in-edge *av*=*au$* can be removed from the in-edges to *v*. The edge *au$* is superfluous since *au* can be reached by backtracking from *ub*. If all in-edges to *v* can be removed by this rule, the previous rule then allows *v* to be removed.

**Algorithm 10** Prune recursal from vertex *i* ending in *$*^*l*^, return *true* if unneeded

The pruning away of superfluous final-completing vertices is not required for the kFM-index to work, but it can reduce the memory required in cases where the number of such vertices is big. As such, depending on the number of final-completing vertices at any point of the processing or merging of kFM-indexes, one may choose to perform this pruning at the end or at some of the intermediary merges.

### Pre-assembly

As a first step of sequence assembly, and a good task for assessing both the resulting de Bruijn subgraph and the efficiency of its use, uniquely determined paths of the graph are determined. This consists of two steps. First, the list of vertices are checked to identify all vertices that have in-degree or out-degree different from one:an algorithm is provided in the Additional file 1. The remaining vertices are simple, non-branching vertices that paths just pass through. Iterating over all branching/ending vertices, all possible paths passing through degree-one vertices are generated. When generating the sequence corresponding to a path, the sequence of the last vertex of the path needs to be found using Algorithm 5, while the rest is determined when backtracking through the in-edges.

The result is a list of non-simple vertices, and a list of all uniquely determined paths between these. An estimate of the number of such paths can be found simply from summing over the in-degrees of all branching vertices, but this may include paths consisting entirely of final-completing vertices. When generating the list of uniquely determined paths, those containing only final-completing vertices are excluded.

## Results

### Memory usage

The main data kept in memory is the *σ*×*n* binary array *η*(*a*,*i*) and the binary flags *f*_*i*_ for marking the end of each vertex group. Direct storage of these in bit-packed arrays requires *σ*+1 bit of information per node, so the total memory for storing the in-edge list and vertex group flags is

(13) $$Mem\left[E,f\right]=n\times (\sigma +1)\text{bit}.$$

For efficient computation of the previous vertex position *ρ*(*a*,*i*), a subset *ρ*(*a*,*i*_*r*_) is permanently stored for positions 0=*i*_0_<⋯<*i*_*ζ*_=*n*. Direct storage of *κ*(*ζa*+*r*)=*ρ*(*a*,*i*_*r*_), where *a*=0,…,*σ*−1 represents the letters, would require *σζ* lg *n* bit since each position requires lg *n* bit. However, decomposing *κ*(*j*)=*u*_*j*_+*qU*_*j*_ where *i*_*r*_−*i*_*r*−1_≤*q*, and storing the *u*_*i*_ and *ΔU*_*j*_=*U*_*j*_−*U*_*j*−1_∈{0,1}, requires only lg*q*+1 bit for each value in *κ*: i.e. *σζ*(lg*q*+1) bit where *ζ*≈*n*/*q* if the *i*_*r*_ are evenly spaced.

In order to efficiently compute arbitrary *U*_*j*_, the Java implementation stores *U*_64*r*_. Since each of these values require lg(*n*/*q*) bit of memory, the total memory requirement for storing the in-edge list, vertex group flags, and the data used to compute the previous vertex position, is

(14) $$Mem\left[\rho_{\text{store}}\right]\approx \frac{\mathrm{n\sigma}}{q}\left(\mathrm{lg}q+1+\frac{\mathrm{lg}(n/q)}{64}\right)\text{bit}.$$

The memory saving construction used to compress *κ* could be repeated for the stored values $\kappa_{r}^{\prime }=U_{\mathrm{\omega r}}$ by writing this as $\kappa_{r}^{\prime }=u_{r}^{\prime }+64U_{r}^{\prime }$, but at an additional computational cost. However, for most practical cases, the term lg(*n*/*q*)/64 is already a very minor part of the memory cost and not worth the computational overhead. By the time *n*>2^64^ becomes an issue, we have probably moved beyond 64 bit computers, in which case increasing the block size of 64 to the higher word size *ω* changes the memory term to lg(*n*/*q*)/*ω* without increasing the computational time.

Under the assumption that *n*<2^*ω*^, where *ω*=64 is the word size of a 64 bit computer, the total memory requirement is

(15) $$Mem\left[E,f,\rho \right]<n\times \left[\sigma +1+\frac{\sigma (\mathrm{lg}q+2)}{q}\right]\text{bit}$$

although there may be some additional overhead depending on how the data is bit-packed into arrays.

For DNA, *σ*=4, which requires 5 bit of data per vertex. However, storing 12 vertices packed into a single 64 bit word leaves 4 unused bits, and so it effectively consumes ≈ 5.333 bit per vertex in the present Java implementation. For stored previous vertex positions, *ρ*_store_, natural step sizes *q* between stored values are *q*=16, 32 and 64, which adds 1.5 bit, 0.875 bit and 0.5 bit of memory usage per vertex, respectively.

### Computational speed

Estimates of computational speeds are based on the assumption that *n*<2^*ω*^ where *ω* is the word size: i.e. *ω*=64 on a 64 bit computer. This means that a number in the range 0 to *n* can be read from memory in one operation: for arbitrarily large *n*, this would require at least (lg*n*)/*ω* operations. It also means single operations can operate on *ω* bits at the same time, although this is largely unexploited by the implementation.

The central algorithm that influences most kFM-index computations is that of computing arbitrary *ρ*(*a*,*i*): Algorithm 2, or the extension of this provided in the Additional file 1 and implemented in the Java program. A subset of the values are stored in a compressed form and can be retrieved in constant time. If every *q*th value is stored, the time required to reconstruct and arbitrary *ρ*(*a*,*i*) will on average be of order *O*(*q*). However, since we will in practice select a fixed *q* of moderate size, which is sufficient to keep memory costs of the auxiliary data at a low level, and a fixed computational time remains even as we let *q* drop towards 1, we may consider the computation of *ρ*(*a*,*i*) to be of constant time.

Algorithm 2 for computing *γ*(*x*,*i*) for any string *x* requires |*x*| calls to *ρ*, and thus has time complexity *O*(|*x*|). Consequently, identifying the interval of vertices with prefix *x* (Algorithm 3) has time complexity *O*(|*x*|). Algorithm 4 for backtracking the graph from vertex *i* along edges provided by the string *x* is essentially the same as the computation of *γ*, just with checks that the edges exist, and also has time complexity *O*(|*x*|). The reverse computation of finding the string representation of a given vertex index, provided in Algorithm 5, requires solving for *ρ*^inv^(*i*) using a binary search, and is thus of time complexity *O*(*k* lg(*nσ*)).

Constructing a kFM-index in memory, provided the memory is sufficient to hold a complete list of *k*-mers from the strings, has time complexity *O*(*Nk* lg*σ* lg*N*) where N=∥S∥ is the total length of the string data. The time is primarily required for sorting the list of in-edges generated from the strings, while construction of the kFM-index from the sorted list is linear in *N*.

Merging two kFM-indexes of sizes *p* and *q* using Algorithm 2 has worst case time complexity *O*((*p*+*q*)*kσ*). This is due to Algorithm 7. The factor *σ* stems from checking sequentially for in-edges and could most likely be replaced by something more efficient should large alphabets be of interest. There is also room for improvement, as detailed in hte Additional file 1, e.g. by reducing the number of computations once conditions like *α*_*B*_=*β*_*B*_ are met.

Construction of a kFM-index in memory by dividing the initial string data into *m* parts, generating kFM-indexes from each, and then merging these pairwise until a single kFM-index remains, has time complexity *O*(*Nkσ* lg(*nm*)) using the present algorithms. At the lowest level, *m* kFM-indexes are generated, each from sorting approximately *N*/*mk*-words, which takes *O*(*Nk* lg*σ* lg(*N*/*m*)) time and is generally fast. During the first roughly lg(*nm*/*N*) rounds of pairwise merges, while the number of partitions is higher than the coverage, the total sizes of the kFM-indexes may still be ≈*N*, and so each round requires time *O*(*Nkσ*). After that, since none of the kFM-indexes have more than *n* vertices, which is the size of the final de Bruijn subgraph, the time complexity drops by a factor of two for each new round of pairwise merges until a single kFM-index remains. The main part of the computations are the first lg(*nm*/*N*) or so rounds of pairwise merges, and so the final kFM-index takes *O*(*Nkσ* lg(*nm*/*N*). As *N* cannot increase without *m* increasing in proportion, since *N*/*m* in-edge *k*-words must be kept in memory, but *m* can increase independently if less memory is to be used, it is more natural to write this *O*(*Nkσ* lg(*nm*)).

### Benchmarking of the Java implementation

The Java implementation has not been optimised for speed: it runs on a single core, and prioritises memory consumption and code generality and readability over speed. However, it can still give a fair indication of the computational speeds, and indicate which are the bottlenecks.

The benchmarks on *E. coli* and simulated data were run on Java 6 under 64 bit Windows 7 on a standard office laptop: Dell Latitude E6320 with Intel Core i7-2620 2.70 GHz CPU and 8 GiB RAM. For *C. elegans* and the soil sample, it was run on a server with more memory and roughly twice the computational speed. The amount of RAM available to Java was set with the option -Xmx. Note that for specifying computer memory, I use IEC prefixes ki-, Mi-, and Gi- which represent powers of 1024, while SI prefixes k-, M-, and G- represent powers of 1000.

All kFM-index constructions from read data added both reads and their reverse complements, discarding pairing information. Quality filtering consisted of removing bases with quality score less than 30, splitting the reads into fragments with higher quality bases. Unless otherwise stated, *k*=23 were used: note that the Java implementation specifies the order *k*−1, i.e. length of the vertex strings. The distance between stored values *ρ*_store_ was *q*=32. This should require 6.2 bit per vertex as the actual memory usage on the data, including 0.33 bit due to the 4 unused bits in the pack of 12 vertices stored in a 64 bit Java long integer, although there would be some additional memory overhead from the program itself.

Memory usage during kFM-index construction was largely determined by the size of partitions, i.e. the maximal number of *k*-mers processed in each partition, which was set to different values to assess the time required to generate kFM-indexes by merging smaller indexes. For runs on the laptop, it was set to process at most 250 M words in each partition, which for *k*≤28 would require 2 GiB of memory with each word using 2×32 bit integers; on the server, the partition size was limited by implementation of the buffer as a Java array with at most 2 G 32 bit values, allowing at most 1 G words in each partition when *k*≤28. However, the peak memory usage reported here includes memory used and released by Java, but not yet garbage collected, and may reflect available memory more than actual use.

#### *E. coli* str. K-12 substr. MG1655

The implementation was evaluated on *E. coli* str. K-12 substr. MG1655 (SRA accession SRX000429, SRR001665, http://www.ncbi.nlm.nih.gov/sra/SRX000429) with 21 M 36 nt reads after discarding pairing information.

The quality filtered graph contained 13.4 M vertices and 13.4 M edges. This was processed in 2 parts and then merged, taking 7.7 minutes, with roughly a fifth of the time spent on merging kFM-indexes. Without quality filtering, the graph contained 81.7 M vertices and 83.7 M edges. For kFM-index construction, this was divided in 5 parts, which were then merged, taking 38 minutes, half of which was spent on merging the kFM-indexes together.

Once the final kFM-indexes had been constructed and the temporary memory freed up, the Java program used 18 MiB holding the quality filtered graph, and 69 MiB holding the unfiltered graph. At startup, before adding data, the Java program used 6–10 MiB of memory.

When given access to 6 GiB of RAM, the peak usage was a bit over 3 GiB. However, by reducing the available memory, peak usage could be reduced to just over 2 GiB, with no substantial change in computational time. The difference is due to memory that has been used and released, but not garbage collected. The main limitation was Java’s ability to allocate the approximately 2 GiB block required to collect and sort in-edges for kFM-index construction.

In the quality filtered graph, 96.3% of the vertices were simple, i.e. had in- and out-degree one. Pre-assembly took 12.9 seconds and produced 427 k uniquely determined paths. In the unfiltered graph, only 89.9% of the vertices were simple, and as a consequence it produced 8.3 M uniquely determined paths using 227 seconds.

#### Simulated read data

Simulated sequence data were generated from two 1 Mnt DNA sequences, which were identical random sequences except from 0.1% random differences, intended to simulate a diploid organism with SNPs. Random 300 k 100 nt reads were generated with error rates 0%, 0.1%, and 1%, intended to represent the true sequences, reads with partial error correction, and raw reads at 30 times coverage.

The reads with no errors resulted in a graph with 2.05 M vertices and 2.05 M edges. This corresponds to 2×1 Mnt plus 22 extra vertices from each strand of the 0.1% SNPs. Accordingly, pre-assembly produced 6358 uniquely determined paths, which corresponds to the sequence between the SNPs and two variants for each SNP.

When the reads were given 0.1% error rate, which may be a realistic error rate after mild error correction, the graph size increased to 3.26 M vertices and 3.31 M edges. Pre-assembly resulted in 153 k uniquely determined paths

Including the full 1% read errors, as is common in uncorrected reads, the size increased to 15.7 M vertices and 16.1 M edges. Pre-assembly resulted in 1.37 M uniquely determined paths.

The time for constructing the kFM-index was 47, 49, and 64 seconds, respectively, for the three cases when enough memory was allocated to process all the data in one part. When the reads were spilt in 6 partitions and then merged, this time increased to 109, 139, and 327 seconds, respectively. The pre-assembly time was proportional to the size of the graph, and was 0.64, 3.7, and 35 seconds, respectively.

#### *C. elegans* str. N2

A kFM-index was generated from 67.6 M 100 nt reads on *C. elegans* str. N2 (SRA accession SRX02594, SRR065390).

With *k* = 23, this resulted in a graph with 255 M vertices and 259 M edges. With reads processed in 8 parts and merged, this took 5.4 hours on the server. Pre-assembly took 7.3 minutes and resulted in 12.8 M uniquely determined paths.

After completion of the kFM-index, the program holding the index used 290 MiB, some of which is unreleased memory after pruning away 61 M final-completing vertices at the end. The buffer used to store each of the 8 partitions took 8 GiB, while peak memory usage was 12 GiB.

#### Soil sample

An additional run was made on a soil sample (SRA accession SRX128885, SRR444039, http://www.ncbi.nlm.nih.gov/sra/SRX128885) with 37 M 76 nt reads.

Quality filtering left 2.59 G 23-mers to be processed, including the reverse complements. The resulting graph consisted of 2.86 G vertices and 2.87 G edges: the increase relative to the number of words added is due to final-completing vertices which represent read suffixes. This took 7.1 hours to generate, processing the data in 4 partitions before merging them. Most of this time was spent merging the kFM-indexes.

After completion, the program only occupied 2.2 GiB of memory, i.e. 6.6 bit per vertex including all overhead, indicating approximately 6% memory overhead relative to the 6.2 bit per vertex required by the data structure. During kFM-index construction, peak memory usage was 14 GiB.

Pre-assembly took 1.08 hours and resulted in 85.6 M uniquely determined paths. These appeared to be mostly from single reads.

## Discussion

### Memory requirements

The memory required to store $E=S^{\left[k\right]}$ as a list of strings would be |*E*|×*k* lg*σ* bit. If the edge set had been an arbitrary subset *E*⊂Σ^*k*^, optimal storage would require

(16) MemE⊂Σk=lgσk|E|bit≈|E|×klgσ−lg|E|ebit

where the approximation assumes that *E* is a sparse subset of Σ^*k*^. This is slightly better than storing *E* as a list of strings since it takes into account that the list of edges is unordered and contains each edge at most once. However, the claim that this is minimal required memory [1], is not strictly true, as $E=S^{\left[k\right]}$ is not an arbitrary subset of Σ^*k*^: it is induced by the sequences in . If most of the strings in  are much longer than *k*, this gives us ample room for reducing the memory usage.

Storing the sequences of , i.e. the data from which $S^{\left[k\right]}$ is generated, requires

(17) $$Mem\left[S\right]=∥S∥\times \mathrm{lg}\sigma \text{bit}$$

where $∥S∥=\sum_{x\in S}|x|$ is the total length of the strings, assuming we do not have to store information about the lengths of the strings: this is true if all strings x∈S have the same length |*x*|=*l*, and a good approximation if the average length of the strings is much greater than the alphabet size.

If the strings of  are very different, in the sense that they do not share *k*-substrings to any particular extent, it will be more memory efficient to store the strings of  directly than storing $S^{\left[k\right]}$, and little can be done to reduce this memory requirement. This was the case with the soil sample data analysed. In this case, the FM-index [11], which is based on the Burrows–Wheeler transform, provides a compact index for representing all substrings of : the Burrows–Wheeler transform requires ∥S∥×lgσbit, i.e. no more than the raw sequences.

When there is substantial overlap between the strings in , there are more compact representations of $S^{\left[k\right]}$. For example, if the strings of  can be assembled into a smaller number of strings, ’, of which the strings of  are substrings, we could use these strings instead to represent $S^{\left[k\right]}$: if each *k*-string in $S^{\left[k\right]}$ is found on average *ν* times, this would approximately reduce the required memory by a factor of *ν*. However, since assembly is a difficult problem, this is not a practical approach. In addition, the strings may not assemble well, e.g. due to read sequencing errors.

One fairly direct way to represent a de Bruijn subgraph, without storing a complete list of all *k*-mers, is to represent edges as pointers between vertices: e.g. an out-edge from a vertex may be stored as a pointer to the target vertex. Storing edges as pointers as well as the letters they correspond to requires |*E*|×(lg|*V*|+ lg*σ*) bit of memory, which can be quite demanding for large graphs. For the 13.4 M graph representing the quality filtered *E. coli* reads, this would be 25.7 bit per vertex: more than four times as much as the kFM-index.

If most vertices are simple, i.e. have only one in-edge and out-edge, the number of pointers required may be drastically reduced by combining them into uniquely determined paths in the graph, as is done in Velvet [12]. Only one pointer would then be required for each such path, which for the quality filtered *E. coli* graph would be 427 k pointers each requiring 18.7 bit, resulting in 2+0.6 bit per vertex for storing the nucleotide and the pointers. By combining vertices representing reverse complements into duplex vertices, the number of vertices and paths is halved, but pointers in both directions must be maintained, making this 2+2×0.6 bit per duplex vertex.

Even if merging non-branching vertices allows the graph itself to be stored more compactly, a map from arbitrary *k*−1-mers to vertices of the graph is required, and storing these pointers would cost at least |*V*|× lg|*V*| bit of memory. A regular hash map would use additional memory to identify *V*⊂Σ^[*k*−1]^, but that is not strictly needed and could be avoided by smart hashing schemes. Still, for the quality filtered *E. coli* reads, a full map for the 13.4 M 22-mer vertices would require at least 23 bit per duplex vertex, and this would increase for larger graphs. If the 22-mers are mapped to the uniquely determined paths rather than to the individual vertices, this could be reduced to 18.7 bit per duplex vertex, which with the 3.1 bit per duplex vertex for storing the graph, still adds up to twice as much as required by the kFM-index, and would increase with the size and complexity of the graph. Many genome assemblers, such as ABySS [13], hash either vertices or edges, and are thus subject to this requirement. In fact, for any data structure that does not throughout make use of the fact that the vertices and edges tend to form long paths, the memory bound of equation (16) applies.

A natural method to compare the kFM-index against is the compressed Burrows–Wheeler transform of the concatenated reads used by SGA [10], due to the similarity between the kFM-index and the Burrows–Wheeler based FM-index. The Burrows–Wheeler transform of the reads would result in runs of identical bases corresponding to the coverage of the reads (unless broken up due to sequencing errors), and the SGA stores each run by its base and run length in a single byte. A naive comparison of memory requirements could be made against the in-edge data of the kFM-index which requires 5 bit per vertex; if most vertices have degree one, the in-edge data can be stored more compactly using only 2–3 bit per vertex since most vertices only require the base of the single in-edge to be stored. However, this is an unfair comparison since the kFM-index only stores the *k*-mers for a specific *k*, while the Burrows–Wheeler transform used by SGA stores all substrings, and thus allows *k*-mer frequencies to be found for all *k*. While the kFM-index specifically stores the de Bruijn *k*-mer subgraph, SGA uses overlap-based assembly and was not made with de Bruijn graphs in mind. SGA also does frequency based read error correction.

Memorywise, the kFM-index is comparable to the probabilistic de Bruijn graph using a Bloom filter [5], which requires approximately 4 bit of data per vertex. However, this can merge *k*-mers with their reverse complements, and this reduces the memory requirement by a factor of two relative to data structures like the kFM-index which has to store both. On the other hand, the Bloom filter is probabilistic, with a risk of introducing false vertices. While it can be used for checking if an arbitrary vertiex is present in the graph, although this requires a lower error rate and thus a little more memory spent per vertex, additional information is required to actually reproduce the graph. Certain removal of false vertices as well information required to reproduce the graph can be added [6], but requires more memory. In comparison, on the same *E. coli* read data, Minia [6] represented 4.7 M “solid” 23-mers (frequency at least 3) using 13.62+0.49 bit per duplex vertex, where each duplex vertex represents both a *k*-mer and its reverse complement. The 0.49 bit were used to store the marking structure required to reconstruct the graph. As such, the amount of memory per vertex is just slightly above the kFM-index, given that kFM-index needs two vertices to represent reverse complements where Minia only needs one.

As can be seen, the different data structures have different strength and weaknesses. Reductions in memory consumption tends to come at the expense of accessibilty of the stored information, computational speed and simplicity. Choice of a suitable data structure for any given problem thus depends on the computational needs, and there is no single best data structure for storing *k*-mer data from high throughput sequencing reads. The kFM-index is particularly made for storing the *k*-mer de Bruijn subgraph representation of sequence reads in a compact manner, yet allowing efficient random access of vertices and edges.

#### Further reduction in memory usage

The main memory usage of the kFM-index is the bit arrays *η*(*a*,*i*) and *f*_*i*_ representing the in-edges and group end flags. These contain *σ*+1 binary values per vertex: 5 bit per vertex for DNA sequence data. For arbitrary de Bruijn subgraphs, little can be done to reduce this substantially.

De Bruijn subgraphs constructed from real sequence data will, however, tend to be dominated by vertices with exactly one in-edge and no other vertices in its vertex group. In this case, for the majority of vertices, the data could simply be summarised by one letter, *a*_*i*_∈Σ, representing the letter of the in-edge to vertex *i*: i.e. *E*_*i*_={*a*} and *f*_*i*_=*true*. This would reduce the required memory from *σ*+1 bit per vertex, and potentially towards lg(*σ*) bit per vertex. The current implementation targets cases with small alphabets, like DNA which has *σ*=4, and little emphasis has been placed on handling large *σ* where lg(*σ*) bit per vertex would make a big difference.

Since not all vertices can be thus represented, there would have to be some way of representing more general vertices as well, which would require both some memory overhead as well as computational overhead. For DNA sequencing reads, the conditions might be met where this approach could be useful, and could possibly reduce the memory consumption for storing the kFM-index by a factor of 2. The entropy of the vertex data, in-edge sets and group end flags, for typical kFM-indexes on DNA sequencing read data tends to be 2 to 2.5 bit per vertex, even for sequence data with read errors included.

#### Effects of read errors

I have not explicitly addressed to problem of read errors. Each read error may result in up to *k*−1 additional vertices, one for each of the *k*−1-substrings containing the read error. If the error rate or coverage is high, this may result in a substantial increase in the number of vertices. For example, if the error rate is *ε*, the chance that a particular *k*-word from a sequence will contain an error is approximately *kε*. If the average coverage is *γ*, this means there will tend to be *γkε* incorrect vertices for each correct vertex: i.e. the number of vertices in the de Bruijn subgraph when incorrect vertices are included will be *n*≈(1+*γkε*)*n*_correct_ where *n*_correct_ is the number of correct vertices. E.g. with 1% error rate, 30 times coverage, and *k*=35, this would make ten times as many incorrect vertices than correct vertices. The simulated read data illustrate this trend.

By also storing vertex counts, the kFM-index could be used for error correction based on *k*−1-mer frequencies. However, there already exist multiple algorithms and applications for excluding infrequent *k*-mers [10,14-18]. Furthermore, much of the advantage of the kFM-index over the compressed Burrows–Wheeler transform of the concatenated reads [10] is lost if the frequency count is to be stored, which is needed for error correction. Hence, the basic assumption has been that the primary usefulness of the kFM-index occurs when the majority of read errors have been removed or corrected in advance, and that error correction is more efficiently done prior to kFM-index construction.

The Java implementation allows filtering by base quality scores, removing bases with low reliability and splitting the sequence accordingly. On a number of benchmarking runs, this simple approach seemed to be able to remove the majority of read errors, and reduce the size of the resulting de Bruijn graph substantially. However, for genome assembly, further error correction would be required.

#### Effect of adding final-completing vertices

The vertices *V* consist of $S^{\left[k-1\right]}$ with vertices added to allow paths from *V*_final_ to *$*^*k*−1^. There may be up to *k*−1 vertices added to *V* for each vertex in *V*_final_. However, as long as |*V*_final_| is small compared to $\left|S^{\left[k-1\right]}\right|$, this has little impact on the memory requirements. We may note that we can always get $\left|V_{\text{final}}|\leq |S\right|$, so if the strings on average are much longer than *k* letters, the contribution of *V*_final_ is likely to be small.

For final-completing vertices to contribute substantially to the memory usage, there would have to be a substantial portion of read ends not found internally in other reads. This could happen for read data consisting of short reads with low coverage. It could also happen if there are frequent read errors at the flanks of the reads which are not filtered out or corrected.

### Computational speed

The central operation in most uses of the kFM-index is the computation of the in-coming vertex position, *ρ*(*a*,*i*). Algorithm 1 gives an implementation whose time complexity on average is *O*(*q*), where *q* is the distance between the stored values *ρ*_store_.

The Java implementation combines algorithm 2 with a similar algorithm provided in the Additional file 1 for computing *ρ*(*a*,*i*) from the next stored value rather than the previous one, and selects the closest stored value, effectively halving the number of steps needed. A good balance between speed and memory usage is then to use *q*=32.

In the Java implementation, the average computational time of *ρ*(*a*,*i*) is linear in *q* as could be expected. In the benchmarking, time per call to *ρ* during large kFM-index was estimated to 75.8+2.8*q* ns. Although some of this is likely overhead related to the merge operations, and the numbers will depend on the computer and implementation, it does give an indication that reducing *q* much below 30 is of limited use. One likely reason for this is the time required for random memory access is high, while repeated accessed to the same block of memory is fast due to caching.

I will treat calls to *ρ* as constant time in the subsequent analyses, although the present implementation does depend on *q*, under the assumption that *q* will remain fixed, typically between 30 and 64, and that in this range the memory overhead of the stored values *ρ*_store_ is moderate.

#### **Low-level parallel computing of *ρ(a,i) *using 64 bit words**

Algorithm 1 works by processing one bit at a time. When doing this, the average number of steps required to compute *ρ*(*a*,*i*) is proportional to the distance, *q*, between the stored values. It is, however, possible to parallell process these bit operations and thus exploit that e.g. a 64 bit processor can process a 64 bit word in one operation.

One such method is described in the Additional file 1, in which the number of steps for processing one word of data is proportional to the alphabet size *σ*. This method requires that the flags indicating if *a*∈ *E*_*j*_ for consecutive *j* are stored in one word, and the same for the group end flags *f*_*j*_, so that the data for an entire interval of *j* positions can be retrieved from memory in a single operation. A replacement for Algorithm 1, provided in the Additional file 1, will then compute arbitrary *ρ*(*a*,*i*) from one word of containing in-edge data for the letter *a* and one word with group end flags, allowing a distance *q*=64−*σ*+1 between stored values: reducing the distance below this has no benefit.

The present Java implementation, however, does not use this approach.

#### Construction of the kFM-index

The main bottleneck at present consist of constructing the kFM-index from the strings. The provided algorithm in which the strings are partitioned into subsets, each subset converted to kFM-indices, and these kFM-indices recursively merged together, introduces substantial overhead in terms of memory and in computational time during construction.

In the FM-index setting, where *n*=*N*, a similar approach would have required time *O*(*n* ln*n*): if we split the data into 2^*r*^ sets, each iteration merges these pairwise using time *O*(*n*) per round, with *r* rounds required. However, for the kFM-index, in the initial steps when the sequences are partitioned, the total number of vertices may be much greater due to *k*−1-words present multiple times in the reads and thus entering into a large number of the subset kFM-indexes. The time consumption may therefore increase by a factor proportional to the coverage and become of order *O*(*Nkσ* lg(*nm*)) where N=∥S∥ is the total size of the sequence data and *m* is the number of partitions into which it is divided.

The algorithm for merging kFM-indexes could most likely be improved substantially, and the Java implementation provided is far from optimal: neither in terms of speed, nor in memory usage. Some minor improvements have been made in the implementation, reducing the number of calls to *ρ* when certain conditions are met (see Additional file 1 for details). However, even with further improvements, the method of partitioning and pairwise merging is inherently slow.

The construction of the kFM-index may be split up and run on separate CPUs; even the final mergers of two kFM-indexes can be split up, e.g. into *σ*^*r*^ different threads based on the first *r* levels of recursion. However, if the reads have high coverage, each of the subset kFM-indexes may already contain many of the same high-coverage vertices, and thus require almost as much memory and processing power as the final kFM-index.

For constructing the Burrows–Wheeler transform and the FM-index, there are more efficient algorithms [19,20] which rely on reformulating the Burrows–Wheeler transform in terms of a shorter sequence over a larger alphabet. These do not easily generalise to the kFM-index. In particular, a *k*-word in the original kFM-index may correspond to multiple different sequence locations, and these may appear as several different *k*-words in the the reformulated sequences. However, I have some hope that the induced sorting approach [19,21] may be adapted, although perhaps not quite as successfully as for the FM-index.

An alternative approach to kFM-index construction in memory is to split the reads up into edges representing the *k*-words of the sequences, and use the disk to help make a sorted list. A simple way to do this can be to partition the read data into parts that are small enough that the in-edge set can be stored as *k*-words and sorted in memory, write each partition to disk as a single file, and then merge these files to construct the final kFM-index. While this may require substantial temporary disk space, particularly for high coverage sequence data, it will most likely be much faster than the in-memory construction of the kFM-index presently implemented. This approach could also be divided between several computers.

#### Use with large alphabets

The algorithms, and the Java implementation, have been written with small alphabets in mind: in particular, DNA with *σ*=4. Technically, they work for general alphabets, although the Java implementation cannot at present handle alphabets with *σ* > 63 since the vertex data are packed into a 64 bit word. However, the routines for merging two kFM-indexes involve iterating over the entire alphabet, adding a time factor *σ* to the merge procedure. For large alphabets, one might store the in-edge data in a more compact form than a bit vector, and could identify the relevant letters without having to check them one by one.

## Conclusions

The kFM-index is a data structure that stores the *k*-words corresponding to the edges of a de Bruijn subgraph in a compact manner, while allowing efficient random access to vertices and edges. The data structure is made compact by avoiding the direct storage of *k*-words and pointers, which often are the main memory expense for storing de Bruijn subgraphs. The vertex and edge information is stored in a direct manner with each line in the data table representing a vertex, and each bit set in the in-edge bit array representing an edge. Thus, the compactness of the kFM-index data structure does not rely on compactification of the graph or compression of the data, and additional compression of the index is feasible.

The presently implemented method for in-memory construction of the kFM-index is uncompetitive for large data sets. However, there are multiple ways in which this could be improved. Also, as with the FM-index used by SGA [10], after the index has been constructed, the user can reuse it to try different assembly options and parameters.

One of the main approaches to de novo genome assembly using high throughput sequencing is to generate the de Bruijn subgraph representing the *k*-mers of the reads, and multiple applications exist already for doing this. For large genomes and in meta-genomics, the memory required for representing the de Bruijn subgraph is one of the limiting factors. The kFM-index could replace existing, more memory demanding, data structures in existing genome assembly applications to allow them to process larger genomes or to run on off-the-shelf hardware where special, high-RAM computers have previously been required.

## Availability

A Java implementation of the data structure and algorithms, together with additional technical documentation of the implementation, are freely available from http://folk.uio.no/einarro/Projects/KFM-index/. Improvements to the implementation, removing memory limitations and introducing parallel processing, and updated benchmarks, are provided on this web site.

## Appendix

### Mathematical approximations

Based on **Stirling’s approximation**, $\ln n!\approx n\cdot \ln\frac{n}{e}$, we can approximate binomials by

(18) lnnx=lnn⋯(n−x+1)x!≲x·lnnx/e

which is a good approximation as long as *x* ≪ *n*. Another bound is

(19) lnnx≤xlnnx+(n−x)lnnn−x≤nln2

where the first inequality follows from nxpx(1−p)n−x≤1 upon entering *p*=*x*/*n*, where nx(xn)x(1−xn)n−x≈n/2πx(n−x) indicating that the inequality is fairly tight, while the second inequality follows from ∑x=0nnx=2n and is only tight for *x*/*n*≈1/2.

### Proofs of results

#### 

**Definition 1. ***For a de Bruijn subgraph G* = (*V*,*E) as defined in equations (1) and (2), we say that a string x corresponds to a path in G if |x|≥ k and all k-substrings of x are contained in E: i.e. x = x*_1_…*x*_*l*_* corresponds to the path e*_*i*_=*x*_[*i*,*i*+*k*−1]_ for *i*=1,…,*l*−*k*+1.* For a string x of any length, we say that x is compatible with G if either x corresponds to a path in G (for |x|≥k) or x is a substring of a vertex v ∈ V (for | x| < k). We write x ∼ G to indicate that x is compatible with G*.

For convenience, we will include *v*_*n*_=*∞*^*k*−1^ in the vertex list, although it is strictly speaking not part of the graph, and apply the convention that *∞*^*k*^ corresponds to a path in *G*. The main effect of this is that the min{*i*∣…} expressions below take the value *n* if criteria for *i* are not otherwise met.

#### 

**Lemma 2. ***Let ρ (a,i) be defined as in equation (6) and γ as in (7). For x a string in* Σ^∗^ or Σ^∗^∘{*$*,*∞*},

(20) $$\gamma (x)=\min\{i|x\leq v_{i}z∼G\text{for some}z\in \Sigma^{*}\}.$$

*For |x| < k, this is just min{i ∣ x ≤ v*_*i*_*}, while for |x| ≥ k it requires a path starting at v*_*i*_ (*unless x ≤ v*_*i*_).

#### 

*Proof.* For the empty string, *γ*(*ε*)=0, making equation (20) true. For *a* ∈ Σ, *γ*(*a*)=*ρ*(*a*,0), and equation (20) is essentially the definition of *ρ*; for *γ*(*$*)=0 the result follows as all *v*_*i*_≥*$*, while for *γ*(*∞*)=*n* only *v*_*n*_≥*∞*. We will then complete the proof by induction on |*x*| using (20) as the induction hypothesis.

For 2≤|*x*|<*k*, write *x*=*ay* where *a* ∈ Σ. By the induction hypothesis, we know that *y*≤*v*_*j*_ when *j*≥*γ*(*y*). Since |*y*|≤*k*−2, this is the same as *y*≤*vj*−, and thus equivalent to *x*=*ay*≤*avj*−. If *x*=*ay*≤*v*_*i*_, then either *v*_*i*_=*avj*− for some *j* in which case *x*≤*v*_*i*_=*avj*− and *i*≥*ρ*(*a*,*j*), or no such *v*_*j*_ exists in which case the first letter *v*_*i*,1_>*a* and *i*≥*ρ*(*a*,*n*). The smallest *i* for which this makes *x*≤*v*_*i*_ in either case corresponds to *ρ*(*a*,*j*) where *j*=*γ*(*y*).

For |*x*|≥*k*, again write *x*=*ay* where we know that *y*≤*v*_*j*_*z*∼*G* when *j*≥*γ*(*y*). This means that *x*=*ay*≤*av*_*j*_*z*=*v*_*i*_*bz*∼*G* for *i*=*ρ*(*a*,*j*) and where the condition *j*≥*γ*(*y*) becomes *i*≥*ρ*(*a*,*j*). □

## Competing interests

The author declares that he has no competing interests.

## Supplementary Material

Additional file 1 Supplementary information on the kFM-index. Click here for file
