# Supplementary material for: Compact representation of k-mer de Bruijn graphs for genome read assembly
Source: BMC Bioinformatics. 2013 Oct 23;14:313. doi: 10.1186/1471-2105-14-313 (PMC4015147; doi:10.1186/1471-2105-14-313)
Supplement: Additional file 1 — Supplementary information on the kFM-index. [file 1471-2105-14-313-S1.pdf]

Supplementary information on the kFM-index

Supplementary algorithms

Previous vertex position computed from the next stored values

In the article, the algorithm for computing $\rho(a, i)$ from the preceeding stored position is presented. The following algorithm is similar, but computes $\rho(a, i)$ from the next stored position.

Algorithm 1 Compute arbitrary $\rho(a, i)$ from next stored value

```
function ρ(a, i)                                          ▷ a ∈ Σ, i ∈ {0, ..., n}
    if i = 0 then return ρ_store(a, 0) end
    j ← ι⁺(i)                                             ▷ stored position j = i_r ≥ i
    p ← ρ_store(a, j)                                     ▷ stored value
    while not f_{j−1} do j ← j − 1 end
    if j < i then return p end
    hasEdge ← false
    loop
        if f_j then                                       ▷ new vertex group
            if hasEdge then p ← p − 1 end
            if j < i then return p end
            hasEdge ← false
        end if
        if a ∈ E_j then hasEdge ← true end
        j ← j − 1
    end loop
    return p
end function
```

The main advantage of this algorithm comes when it is combined with the original one, which computes $\rho(a, i)$ based on the previous stored value, picking either algorithm based on which has the closer stored value. This reduces the average distance to the stored value by a factor of two, thus allowing q to be twice has high without additional computational costs.

Improved PreMerge function

The PreMerge function is presented in the article in a simple, straight forward way. However, there are a number of special cases in which the computations can be done more efficiently. When $\alpha_B = \beta_B$ is encountered, what remains is to insert the interval of vertices $[\alpha_A, \beta_A\rangle$, which does not require computing all four ρ values. Another similar case is when the A and B intervals both contain exactly one vertex.

The following is a version of PreMerge where these two cases are handled separately.

Algorithm 2 Prepare merge: recurse over A intervals

```
function PreMerge(l, α_A, β_A, α_B, β_B)
    if α_A = β_A then exit end                            ▷ A interval is empty
    if α_B = β_B then                                     ▷ B interval is empty
        for i_A = α_A to β_A − 1 do PreInsertA(l, i_A, α_B) end
        exit
    end if
    if α_A + 1 = β_A and α_B + 1 = β_B then               ▷ since vertices
        PreMergeSingles(l, α_A, α_B)
        exit
    end if
    if l = 1 then group[α_A + α_B], ..., group[β_A + β_B − 2] ← true end    ▷ vertex group
    for a = 0 to σ − 1 do                                 ▷ prefixing letter
        PreMerge(l − 1, ρ_A(a, α_A), ρ_A(a, β_A), ρ_B(a, α_B), ρ_B(a, β_B))
    end for
end function
```

The case $l = 0$ will always be handled by one of these rules and handled in the either of the two new functions.

Algorithm 3 Prepare merge: insert individual A vertices

```
function PreInsertA(l, i_A, i_B)
    if l = 0 then
        isA[i_A + i_B] ← true
        exit
    end if
    for a ∈ Σ do
        if a ∈ E^A_{i_A} then preInsertA(l − 1, ρ_A(a, i_A)) end
    end for
end function
```

Algorithm 4 Prepare merge: merge single vertices

```
function PreMergeSingles(l, i_A, i_B)
    if l = 0 then
        isA[i_A + i_B] ← true
        same[i_A] ← true
        exit
    end if
    if l = 1 then group[i_A + i_B] ← true end
    for a ∈ Σ do
        if a ∈ E^A_{i_A} then
            if a ∈ E^B_{i_B} then PreMergeSingles(l − 1, ρ_A(a, i_A), ρ_B(a, i_B)) end
            else PreInsertA(l − 1, ρ_A(a, i_A), ρ_B(a, i_B))
        end if
    end for
end function
```

In both functions, the data required to make the computations, like E_i, can be looked up in one operation, which saves a little time. The most critical aspect, however, is that the number of calls to ρ is reduced.

Algorithm for generating uniquely determined paths

The first step in finding the uniquely determined paths is to identify the non-simple, or complex, vertices: i.e. those that do not have in-degree and out-degree both equal to one.

Algorithm 5 Prepare merge: merge single vertices

```
function NonSimpleVertices
    mark ← array[n](false, . . . , false)
    E_group ← ∅; E_dual ← ∅
    for i = 0 to n − 1 do
        E_dual ← E_dual ∪ (E_group ∩ E_i)
        E_group ← E_group ∪ E_i
        if f_i then
            for a ∈ E_dual do mark[ρ(a, i)] ← true end
            E_group ← ∅; E_dual ← ∅
        end if
    end for
    return mark
end function
```

Starting with the list of non-simple vertices, for each in-edge to a non-simple vertex, start backtracking until another non-simple vertex is reached. For each such in-edge, that path is uniquely determined, and thus all uniquely determined paths are generated. Some of these paths math start and end in final-completing vertices, and thus represent a suffix of length less than k: these may be excluded when the string representation of the path has been produced.

Parallel bit-processing for computing previous vertex position

The algorithm presented in the article, and implemented in the Java program, uses single bit operations to compute $\rho(a,i)$ from stored values $\rho_{\text{store}}(a,i_r)$ where $0 = i_0 < \cdots < i_\zeta = n$ are the stored positions with steps at most q.

For a computer with word size ω, i.e. $\omega = 64$ for 64 bit computers, it is possible to process ω bits of information in parallel. This can provide substantial speed up relative to single bit operations: both because of the number of computational operations, and by having the data required for the computations packed into single words rather than read from memory one vertex at a time.

The present Java implementation stores the $\sigma + 1$ bits of information per vertex in one block, which for DNA with $\sigma = 4$ results in 12 such blocks being stored in each 64 bit word. The present implementation reads one vertex at a time without exploiting that fact consecutive vertices are likely to be stored in the same word.

For efficient parallelisation of bit operations, it is better to reorganise the data storage so that the ω bits of data that should be parallel processed are stored in a single word. For this, we use $\sigma + 1$ separate words for storing data for each of the letters and for the vertex group end flags. For computing $\rho(a,i)$, we would then need to read one word containing data for $\eta(a,j)$ for some interval of j that contains i, and one word with the corresponding vertex group data f_j.

In order to ensure all vertex groups are stored within a single word, not split across words, some margin needs to be added: instead of blocks of length ω, blocks of length $\nu = \omega - \sigma + 1$ are used. We then store data in words $\xi^{(a,r)} = \xi^{(a,r)}_{\omega-1} \ldots \xi^{(a,r)}_0$ and $\phi^{(r)} = \phi^{(r)}_{\omega-1} \ldots \phi^{(r)}_0$, where $\xi^{(a,r)}_j$ and $\phi^{(r)}_j$ represents the individual bits:

$$\xi^{(a,r)}_j = \eta(a, r\nu + \omega - \sigma - j),\ \phi^{(r)}_j = f_{r\nu+\omega-\sigma-j} \text{ where } a \in \Sigma,\ j = 0, \ldots, \omega - 1 \tag{1}$$

where any bits outside the range of definition are set to 0. This makes

$$\xi^{(a,r)} = \rho(a, r\nu - \sigma + 1) \ldots \rho(a, r\nu) \ldots \rho(a, r\nu + \nu - 1), \quad \phi^{(r)} = f_{r\nu-\sigma+1} \cdots f_{r\nu} \cdots f_{r\nu+\nu-1} \tag{2}$$

so that for any set group end flag $f_{r\nu+j}$ with $j \geq 0$ we ensure the entire vertex group is included in $\xi^{(a,r)}$. When computing $\rho(a,i)$, we select $r = \lfloor i/\nu \rfloor$ to ensure that $i = r\nu + j$ for some $j \in \{0, \ldots, \nu - 1\}$.

The core algorithm takes the in-edge flags $\xi^{(a,r)}$ and vertex group end flag $\phi^{(r)}$ and computes a word χ where set bits represent the vertex groups that contain and a in-edge. Starting with the stored value $\rho_{\text{store}}(a, (r+1)\nu)$, we can then subtract the number of vertex groups that contain an a in-edges, from the group containing i to the end of the block.

Algorithm 6 Whole word parallel computation of $\rho(a,i)$ from next stored value

```
function ρ(a, i)                                      ▷ a ∈ Σ, i ∈ {0, ..., n}
    r ← ⌊i/ν⌋;   j ← i mod ν                          ▷ rν + j = i
    ξ ← ξ^(a,r);   φ ← φ^(r);   φ̄ = not φ             ▷ φ̄ is the bitwise complement of φ
    χ ← ξ and φ                                       ▷ process last vertex in each group
    loop σ − 1 times                                  ▷ ensure all other vertices in group are processed
        ξ ← (ξ and φ̄) shift −1                        ▷ delete processed vertices and shift one position
        χ ← χ or (ξ and φ)                            ▷ process next vertex in each group
    end loop                ▷ χ now contains bits set for each group containing an a in-edge
    p ← bitcount(χ shift +(σ − 1 + j))                ▷ count groups starting at position j within block
    return ρ_store(a, (r + 1)ν) − p
end function
```

Note that the logical operations are performed bitwise, and the shift operator χ shift q shifts the bits of χ q positions to the reft or $-q$ positions to the right: e.g. χ shift $-1 = 0\chi_{\omega-1} \ldots \chi_1$. The bitcount function exists as a single word operation with low-level implementation in e.g. Java.

For faster memory access, we suggest storing the ξ and ϕ data so that $\xi^{(a,r)}$ and $\phi^{(r)}$ for a single r are located on the same area of memory: e.g. $M_{(\sigma+1)r+a} = \xi^{(a,r)}$ where $a = 0, \ldots, \sigma - 1$ represents the letters and $M_{(\sigma+1)r+\sigma} = \phi^{(r)}$. The reason for this is that CPU optimisations like prefetching and caching can make access to words stored successively in memory much faster than random access.

For DNA sequences processed on a typical 64 bit computer, $\sigma = 4$ and $\omega = 64$, and the block size $\nu = 61$ can be used. The maximal block size referred to in the article then becomes $q = 61$, and so this requires $(4+1) \times 64/61$ bit of information for storing the main data, and $4 \times 8/61$ bit for ρ_{store} since $\lg q + 1 + \lg(n/q)/64 < 8$. In total, that is 5.77 bit per vertex.

The computational complexity of computing arbitrary $\rho(a,i)$ is $O(\sigma)$ due to the need to cover the maximal number of vertices in a vertex group. As in the article, this assumes that $n < 2^\omega$: when this limitation is broken, the number of computational steps required whenever any position value is involved will increase by a factor of $(\lg n)/\omega$. In that respect, the computational complexity of algorithm 6 is $O(\sigma + (\lg n)/\omega)$ as n increases. The bitcount function has complexity $O(\lg \omega)$, but will still tend to be a single CPU operation. Thus, apart from these technical assumptions, the complexity remains $O(\sigma)$ which given a fixed σ is constant time.

However, the apparently significant improvement from a time complexity $O(q)$ algorithm, presented in the article and implemented in the Java program, to a complexity $O(\sigma)$ algorithm may be less significant than the numbers indicate for moderately sized q. As noted above, random memory access is much slower than sequential memory access, and although the complexity increases with increasing q, benchmarking of the implementation indicated limited gain from reducing q much below 32. If we compute a baseline time required by extrapolating to $q = 1$, i.e. where all of ρ is stored, we find that the time used with $q = 32$ is roughly twice the baseline time, and at $q = 64$ it is three times the baseline time. This may in part be due to the time required for random memory access, and will most likely not be reduced by improvements in the algorithm. Thus, the bit-parallelised algorithm may be expected to be three times as fast as the present one for $q \approx 64$, and twice as fast than $q \approx 32$ although with slightly less memory consumed. As the algorithm requires a different organising of the data and reduced generality, it has not been implemented in the Java program.
